# Influence of nitrate-containing arugula juice on nitrate-reducing oral bacteria and periodontopathogens in smokers’ biofilm

**DOI:** 10.3389/fdmed.2025.1545479

**Published:** 2025-05-09

**Authors:** Boy M. Bachtiar, Irene E. Rieuwpassa, Heni Susilowati, Retno Indrawati, Citra F. Theodorea, Turmidzi Fath, Endang W. Bachtiar

**Affiliations:** ^1^ Department of Oral Biology, Faculty of Dentistry, Universitas Indonesia, Jakarta, Indonesia; ^2^ Oral Science Research Center, Faculty of Dentistry, Universitas Indonesia, Jakarta, Indonesia; ^3^ Department of Oral Biology, Faculty of Dentistry, Universitas Hasanuddin, Makassar, Indonesia; ^4^ Department of Oral Biology, Faculty of Dentistry, Universitas Gadjah Mada, Yokyakarta, Indonesia; ^5^ Department of Oral Biology, Dental Medicine Faculty, Universitas Airlangga, Surabaya, Indonesia

**Keywords:** arugula, biofilm, nitrate-reducing bacteria, periodontopathogens, nitrate-associated genes

## Abstract

Green leafy vegetables such as arugula are rich in nitrates that support oral health, yet their effects on oral microbial balance, especially in smokers, remain unclear. This study evaluated the survival and activity of nitrate-reducing bacteria (NRB; *Veillonella* spp. and *Rothia* spp.) in biofilm exposed to nitrate-containing arugula juice (3.25 or 6.25 μM). The proportions of NRB were compared with periodontopathogens (*Porphyromonas gingivalis* and *Fusobacterium nucleatum*). Using quantitative real-time PCR (qPCR), we assessed bacterial survival and transcription of nitrate reductase genes (*narG* and *napA*) in biofilm from smokers and non-smokers. The results revealed that nitrate-containing arugula juice increased NRB bacteria abundance while reducing periodontopathogen growth. A higher level of nitrate (6.25 μM) increased nitrate reductase expression. Prolonged exposure (9 h) sustained the growth-promoting effect on *Rothia* spp. These results suggest that non-smokers have more nitrate-reducing bacteria in their biofilm, which promotes oral microbial balance. Thus, smokers might be advised to consume nitrate-containing arugula juice to promote NRB, which may have health benefits.

## 1 Introduction

The human oral cavity hosts a complex microbial community that maintains health when in balance (symbiosis), but can lead to disease when disrupted (dysbiosis) (1). Oral dysbiosis is associated not only with oral issues such as caries and periodontal diseases but also systemic conditions, including diabetes, cancer, and cardiovascular disease (2, 3). Maintaining a healthy oral microbiota is therefore critical for overall health.

Cigarette smoking, a major public health concern, disrupts the oral microbiota by reducing diversity and promoting an anaerobic environment (4, 5). This disruption often occurs before clinical symptoms arise and is linked to an increased risk of systemic diseases (6). Although smoking is prevalent in Indonesia, little is known about its specific effect on the oral microbiota in this population, particularly on nitrate-reducing bacteria (NRB), which play a role in maintaining oral health. Understanding these effects could provide valuable insight into related oral dysbiosis in Indonesians.

Nitrate-rich vegetables, such as arugula (*Eruca sativa*), have gained attention for their potential to support oral health by promoting beneficial nitrate-reducing bacteria. These bacteria convert dietary nitrate into nitrite and then nitric oxide, an antimicrobial agent that helps prevent dysbiosis (1, 7). However, the role of arugula juice in mitigating the effects of smoking on oral health remains unclear. The aim of this study was to explore whether nitrate-containing arugula juice affects smokers’ salivary biofilm, specifically how it can promote nitrate-reducing bacteria and prevent dysbiosis. By exploring this, this study seeks to develop new strategies for managing smoking-related oral health challenges.

## 2 Materials and methods

### 2.1 Participants

The saliva donors included 12 smokers and 12 non-smokers aged 20–35 years, with a balanced male sex distribution (60%–75% in each group). The participants had good general health, absence of systemic disease, at least 20 teeth with no active caries (good cavity fillings were acceptable), no impacted teeth, no inflamed third molars, and no teeth with root canal treatment or periapical lesions.

To mitigate potential confounding factors that could influence the baseline oral microbiome composition and subsequently *in vitro* biofilm formation, we implemented strict inclusion and exclusion criteria. All participants, both smokers and non-smokers, were selected based on having good oral hygiene, as assessed by the simplified oral hygiene index (OHI-s) category (8), and plaque index (PI) < 1 (9, 10). Exclusion criteria included antibiotic or oral antiseptic use within the last month, oral protheses, orthodontic appliances, gingivitis, or chronic periodontal disease. Smokers were defined as those smoking at least one cigarette daily, while non-smokers had no history of tobacco use.

### 2.2 Saliva collection

It was suggested that the individuals adhere to their regular daily dietary routine. No particular dietary or drinking instructions were offered (11). Unstimulated saliva was collected in the morning after fasting for 1 h. After letting their saliva gather for approximately a minute, participants spat 2–3 ml of saliva into sterile tubes. Samples were stored on ice and frozen at −80°C until analysis. Ethical approval was granted by the Faculty of Dentistry, Universitas Indonesia (Protocol number: 010580724).

### 2.3 Nitrate estimation in saliva

Salivary nitrite was quantified using the Griess reaction (12). Test samples (100 µl) were mixed with Griess reagents (Promega Corporation, Madison, WI, USA), incubated for 10 min at room temperature, and absorbance was measured at 540 nm using an ELISA reader.

### 2.4 Preparation of arugula juice

Fresh arugula leaves were washed, fried, and stored at −4°C before use. A blend of 100 g leaves and 100 ml of cold phosphate buffer saline (PBS) was homogenized, centrifuged (12,000 rpm, 15 min, 4°C), filtered (0.22 µM), and stored at 4°C for up to 24 h (13).

### 2.5 Biofilm assay

Pooled saliva from smokers and non-smokers was centrifuged, and pellets were resuspended in PBS. Saliva (30 µl, containing bacteria/10^8^ CFU ml) was mixed with arugula juice (30 µl, nitrate concentrations of 3.25 or 6.25 µM) and 40 µl of brain heart infusion (BHI) broth and then inoculated into 96-well plates. Biofilms grown without nitrate served as controls. Plates were incubated at 37°C aerobically and anaerobically (microaerophilic) using a gas mixture (H_2_ 10%, CO_2_ 10%, and N_2_ 20%), incubated for 5 and 9 h, and biofilm bacteria were quantified using quantitative real-time PCR (qPCR).

### 2.6 PH and nitrate measurement in biofilms

Biofilm pH was assessed using a pH indicator strip. Our aim was to ascertain whether the pH was above or below 6 (14).

### 2.7 qPCR analysis

DNA and RNA were extracted from biofilm cells after removing extracellular DNA and non-viable cells (14). The Qubit assay kit with a Qubit fluorometer (Invitrogen) was used to measure the quantity and quality of the DNA and RNA. We measured the amount of bacteria's target DNA and mRNA transcription of nitrate-associated genes (*narG* and *napA*) in the biofilm using the SYBR green I binding dye and the particular primers listed in Table 1. The qPCR was carried out using the PCR procedure as previously reported (14) in LightCycler-96 (Roche). The abundance of each targeted bacteria was determined using the relative proportion to total bacteria (15, 16). Relative gene expression was calculated using the 2^−ΔΔCt^ method, with nitrate-free biofilms acting as the control.

**Table 1:** Primers used in this study.

No	Primers	Sequence (5ʹ—3ʹ)		Reference
1	16S rRNA	F	AGAGTTTGATCMTGGCTCAG	(46)
		R	CGTATTACCGCGGCTGCTGG	
2	*Porphyromonas gingivalis*	F	ATAGTAGCGTGTCCGGCTTC	(47)
		R	ATCGTAGGCGGATTGGAGA	
3	*Fusobacterium nucleatum*	F	GCGCGTCTAGGTGGTTAT	(48)
		R	TAGTTCCGCTTACCTCTCCAG	
4	*Rothia mucilaginosa*	F	ACACGTGAGTAACCTACCCTT	(49)
		R	GCAGGTACCGTCAATCTCTC	
5	*Rothia dentocariosa*	F	GGGTTGTAAACCTCTGTTAGCATC	(49)
		R	CGTACCCACTGCAAAACCAG	
6	*Veillonella atypica*	F	GTGCTGCAGAGAGTTTGATCCTGGCTC	(50)
		R	CACGGATCCTACGGGTACCTTGTTACG	
7	*Veillonella parvula*	F	AGCACTTTGGGTGGGAACTC	(51)
		R	GTACGTGTGTAGCCCCAGGTC	
8	*NarG*	F	CAGGCGGCCGCGGATCATCGGG	(52)
		R	CAGCAGACCGACTACCCGCGC	
9	*NapA*	F	CAGCCCATCGGCTCGTC	(52)
		R	AGAACGGCGAGTTCACG	

### 2.8 Statistical analysis

Data were analyzed using GraphPad PRISM 10. Differences between groups (e.g., nitrate levels, bacterial abundance, and gene expression) were assessed using Kruskal–Wallis ANOVA or Student's *t*-test, with significance set at *p* < 0.05.

## 3 Results

### 3.1 Salivary nitrate–nitrite levels (smokers vs. non-smokers)

**Figure 1:**
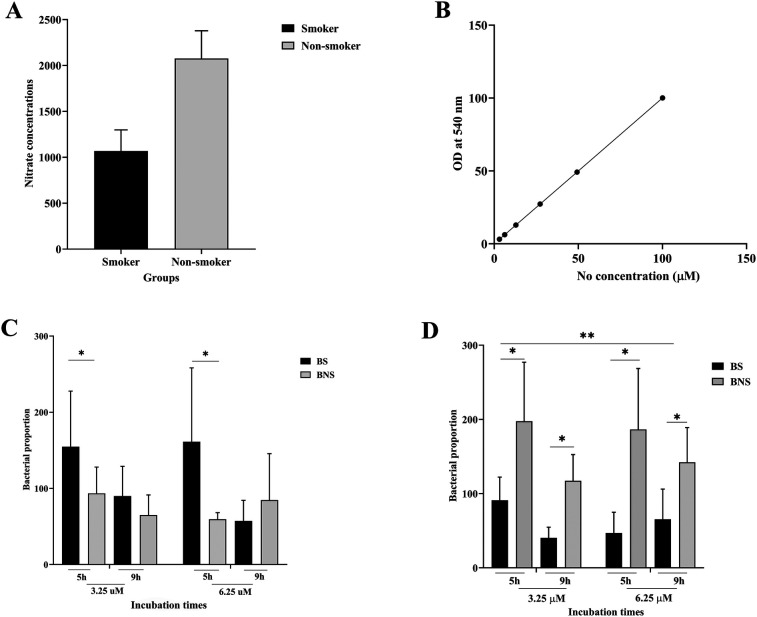
Comparison of nitrate-nitrite levels found in saliva and salivary bacterial counts from smokers (BS) and non-smokers (BNS) in biofilm. Salivary nitrate concentrations in non-smokers were substantially higher than in smokers **(A)**, and nitrate concentrations were calculated using a standard curve **(B).** The bacterial proportions, which were evaluated in both aerobic **(C)** and anaerobic **(D)** conditions, were affected by varying arugula nitrate concentrations and incubation times. * indicates statistical significance (*p* < 0.005).

The comparison of salivary nitrate–nitrite levels between the two groups (smoker and non-smoker) is summarized in Figure 1A. A Griess reaction standard curve (Figure 1B) validated the accuracy of the nitrate–nitrite measurement. This demonstrates that the assay was sensitive across a wide range of concentrations (0–150 µM), ensuring reliability in detecting the differences observed in smokers and non-smokers. We discovered that smoker participants had significantly lower nitrate–nitrite levels than non-smoking participants (*p* < 0.05).

### 3.2 Impact of arugula nitrate on bacterial biofilms

As shown in Figures 1C,D, in aerobic conditions, bacteria from smokers (BS) showed higher abundance than those from non-smokers (BNS) at 5 h with both nitrate concentrations (3.25 and 6.25 µM). At 9 h, the growth of BS bacteria was no longer increasing but was still higher than BNS (with nitrate at 3.25 µM). The reverse was found in anaerobic conditions, as BNS consistently showed higher bacterial abundance under all nitrate concentrations. However, this increase diminished over time, as we observed at 9 h time period.

### 3.3 The impact of arugula-derived nitrate on nitrate-reducing bacteria (*Rothia* spp. and *Veillonella* spp.)

**Figure 2:**
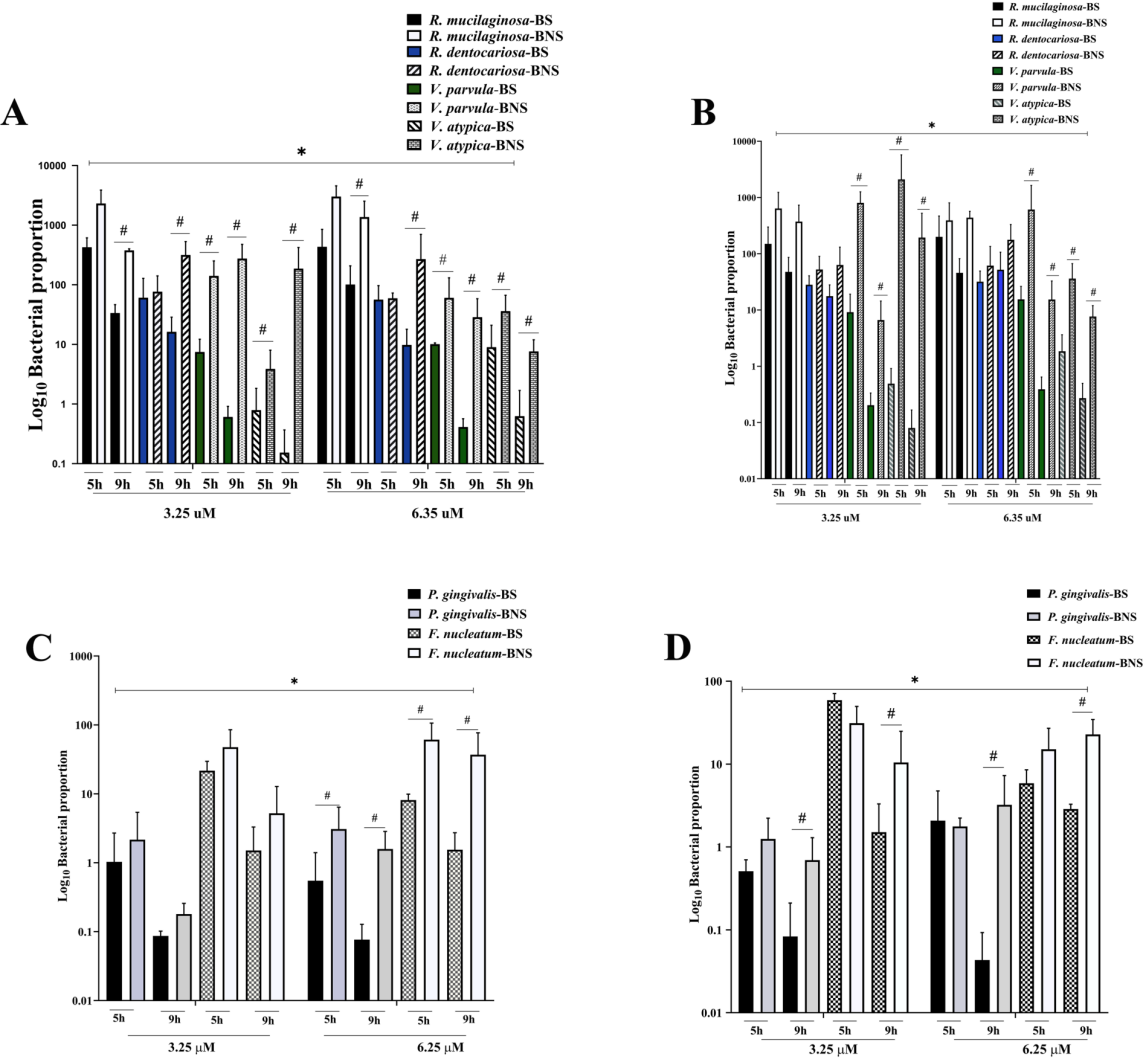
The assessment of nitrate-reducing bacteria and periodontopathogens in the biofilm assay. The bar graph shows that the numbers of nitrate-reducing bacteria **(A,B)** and periodontopathogens **(C,D)** varied significantly in both aerobic **(A,C)** and anaerobic **(B,D)** settings. The impact of environmental conditions on microbial communities was highlighted by the significant difference in nitrate-reducing bacteria proportions depending on arugula nitrate concentrations. However, the periodontopathogen counts showed how incubation time affected the growth of these bacteria, indicating that the biofilm environments have an impact. The results indicated the relationship between smoking status, specific experimental conditions, and different microbiological profiles. * indicates that nitrate concentration differences were statistically significant *(p* < 0.05). # denotes a statistically significant difference between BS and BNS (*p* < 0.05).

As shown in Figures 2A,B, at 9 h and in aerobic conditions, there was a significant growth of *Rothia* and *Veillonella* spp. in the BNS samples, regardless of the nitrate concentrations (3.25 and .25 μM). After 5 h of incubation, a higher abundance of *Rothia* spp. was found in both conditions, irrespective of the sample's source (BS or BNS), and this remained so at 9 h for all arugula nitrate concentrations. We found that the increased proportion of *Veillonela* spp. was influenced by the duration of incubation, but their numbers were still lower than those of *Rothia* spp.

When comparing the NRB species, *R. mucilaginosa* showed robust growth, especially in biofilm derived from the BNS group, with significant variations. Furthermore, there were significant variations in *V. parvula* between the BS and BNS groups, with BNS samples exhibiting greater proportions under both nitrate concentrations. The amount of *V. atypica* in biofilm derived from BNS samples was significantly higher than that in biofilm derived from BS samples under all conditions (aerobic and anaerobic, and at 5 and 9 h).

### 3.4 Effect of arugula nitrate on periodontopathic bacteria (*P. gingivalis* and *F. nucleatum*)

We further investigated potential changes in periodontopathogens. As shown in Figure 2C, in aerobic conditions and at 5 h, the presence of arugula nitrate (3.25 μM) significantly suppressed the growth of both *P. gingivalis* and *F. nucleatum* in biofilms, irrespective of whether the bacteria originated from BS or BNS samples. Yet the growth inhibition occurred in a time-dependent manner. By 9 h, the growth suppression was more significant in the BS samples. Moreover, under anaerobic conditions, both nitrate concentrations (3.25 and 6.25 μM) were effective in reducing the growth of *P. gingivalis* and *F. nucleatum*, particularly after 9 h. The reduction was again more significant in the BS samples (Figure 2D).

### 3.5 The impact of arugula-derived nitrate on transcription of *narG* and *napA*

**Figure 3:**
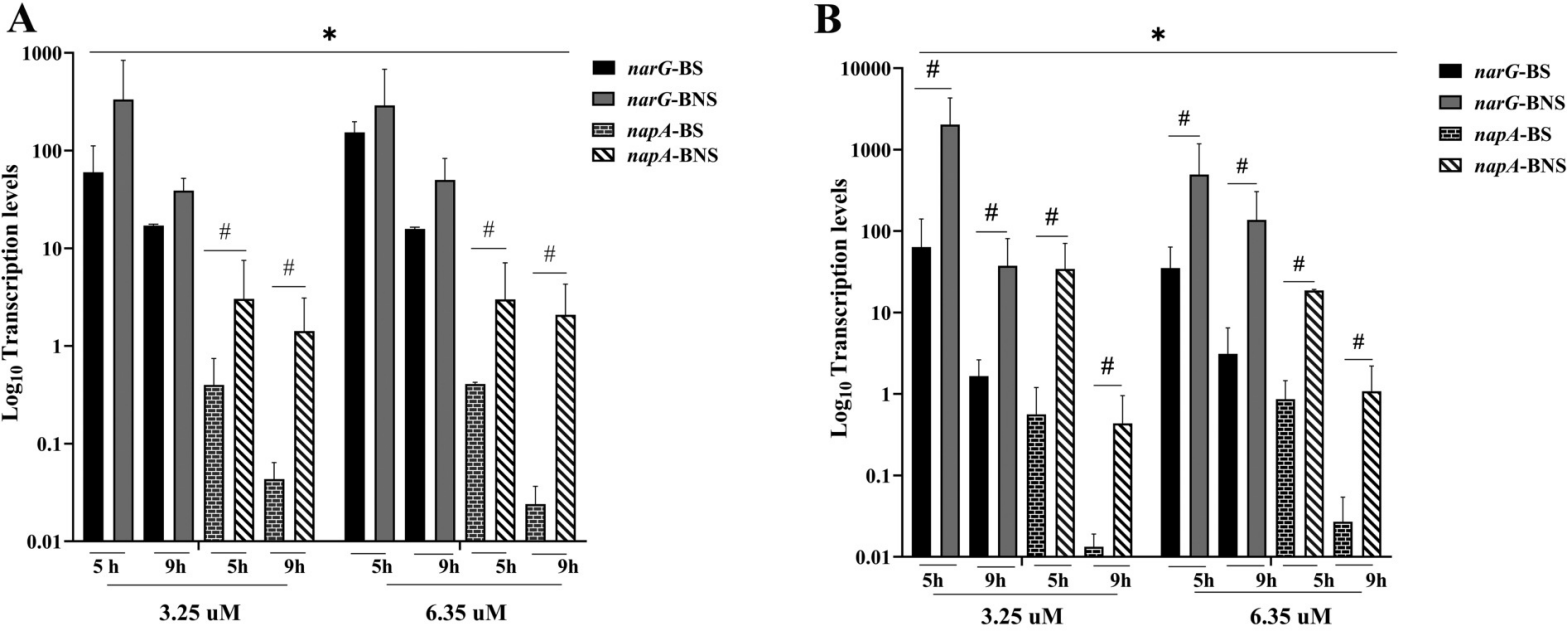
Nitrate-associated genes (*narG* and *napA*) transcription levels in biofilm from non-smokers (BNS) and smokers (BS). (**A**) Gene expression variations between groups under aerobic conditions for 5 and 9 h at different nitrate concentrations (3.25 and 6.25 µM). (**B**) shows the influence of environmental variables on gene transcription levels under anaerobic conditions with similar nitrate concentrations and incubation times. The findings show a significant difference in *narG* and *napA* expression, indicating unique metabolic capabilities according to experimental conditions and smoking status. * indicates that nitrate concentration differences were statistically significant (*p* < 0.05). ^#^ indicates a significant difference between BS and BNS (*p* < 0.05).

In comparison to the mRNA expression of the nitrate reductase-associated genes (*narG* and *napA*), the results demonstrated that under aerobic conditions (Figure 3A), transcription levels of both genes were consistently higher than in anaerobic conditions (Figure 3B). In both aerobic and anaerobic conditions, BNS frequently showed higher gene expression than BNS. In almost all conditions, *narG* exhibited greater expression levels than *napA*. Additionally, gene expression was higher at higher nitrate levels (6.25 µM) than at 3.25 µM, especially for *narG*. For all conditions, transcription levels typically decreased with time (5–9 h), especially for *napA*.

## 4 Discussion

The results of this study highlight the complex interplay between bacterial origin (smokers vs. non-smokers), environmental conditions (nitrate, oxygen), and the expression of nitrate-associated genes. We found that smoking had a significant impact on salivary nitrate-nitrite levels. This is in line with a previous study that found that smokers who practice adequate oral hygiene may have a less efficient nitrate reduction pathway than non-smokers (17, 18). Earlier studies reported that smoking mostly affects the oral environment by disturbing its balance, which results in dysbiotic oral communities and elevated oxidative stress (19–21). Vegetables high in dietary nitrate could be incorporated into the biofilm environment in order to restore the imbalance (22).

The present investigation demonstrated that the biofilms from smokers and non-smokers respond differently to the availability of oxygen and arugula nitrate, while the pH of the biofilms’ spent media remained constant (in the range of 5.5–7) in both anaerobic and aerobic conditions, regardless of nitrate concentrations (not shown). When smokers’ and non-smokers’ bacteria were compared, we observed that under aerobic conditions, BS biofilms initially showed greater abundance under both nitrate concentrations (3.25 and 6.35 μM). This suggests that smokers’ bacteria initially had a higher potential for aerobic nitrate use. This could be because the smokers’ oral microbiome had changed to support nitrate metabolism, possibly as a result of stresses associated with smoking (5, 23). However, this advantage diminished over time, as we observed at the 9 h time period. This could be due to competitive interactions, resource depletion, or shifts in community dynamics (24).

In contrast, in an anaerobic environment, BNS displayed higher bacterial abundance at both nitrate concentrations during the early time points (5 h). This result suggests that under anaerobic conditions, BNS may maintain nitrate-reducing capacity more effectively, possibly reflecting adaptations to low-oxygen niches typical of a healthy oral environment, which smoking disrupts through oxidative and metabolic shifts (25). This finding aligns with existing research suggesting nitrate metabolism can stimulate eubiosis in individuals without periodontitis (26), as we found in our smoker participants with good OHI. Further investigation is needed to understand the underlying mechanisms and potential implications for smokers’ oral health.

Concerning nitrate-reducing bacteria (*Rothia* spp. and *Veillonella* spp.), our results are aligned with earlier studies demonstrating that the NRB is necessary for the oral nitrate–nitrite–nitric oxide (NO) pathway (27, 28). We found that the NRB in BNS samples showed significant growth at 9 h in aerobic biofilm conditions, regardless of nitrate concentrations (3.25 or 6.35 µM). By comparing their growth patterns, we found that the NRB increased over time in all biofilm conditions. *Rothia* spp., particularly *R. mucilaginosa*, showed robust growth, especially in biofilm from non-smokers. *Veillonella* spp. also increased, but to a lesser extent. These findings highlight the potential of arugula nitrate to promote the growth of beneficial nitrate-reducing bacteria, which may contribute to a healthier oral environment (29). This suggests that in our *in vitro* setting, aerobic environments combined with nitrate availability favored the metabolic activity and growth of these bacteria. In anaerobic conditions, *Rothia* spp. showed a consistently higher abundance after 5 h of incubation and sustained growth at 9 h, independent of the sample origin (BS or BNS). This indicates that when the biofilm matures, the microenvironment is conducive to nitrate respiration by aerobic or facultative anaerobic bacteria (30), such as *Rothia* spp. Thus, our results indicated that adding nitrate from arugula juice provides *Rothia* spp. with an exogenous source of a crucial substrate for anaerobic respiration. The finding may be explained by the fact that the bacteria may thrive in anaerobic environments because nitrate acts as an alternative electron acceptor, allowing these organisms to efficiently carry out anaerobic respiration (31).

Conversely, our data revealed that *Veillonella* spp. grew in a way that was significantly impacted by the period of incubation, but their numbers were consistently lower than those of *Rothia* spp. Therefore, while responsive to nitrate, *Veillonella* spp. appears to grow more slowly or be less adapted compared to *Rothia* spp., particularly under anaerobic conditions. This suggests that *Veillonella* spp. may require longer adaptation or exhibit slower growth under these conditions. This finding highlights the species-specific kinetics of nitrate metabolism (32). Nevertheless, a dose-dependent response was demonstrated by the fact that the proportions of *Rothia* and *Veillonella* species were frequently larger in the 6.25 µM nitrate concentration compared to the 3.25 µM concentration. This suggests that high nitrate levels in arugula juice promote the growth or activity of NRB, which are environmentally adapted to both aerobic and anaerobic settings (28). Thus, when nitrate is exposed, *Rothia* spp. and V*eillonella* spp. seem to have a selection advantage. *Rothia* spp. operates efficiently under aerobic and anaerobic conditions, while *Veillonella* spp. prefers anaerobic conditions for optimal activity. The results highlight the metabolic versatility of *Rothia* spp. and how artificial manipulation of nitrate levels and oxygen availability in controlled experiments can influence microbial growth patterns. This aligns with broader studies on biofilm ecology and bacterial adaptability to nutrient and oxygen gradients (33). Taken together, our study suggests that the significant growth of nitrate-reducing bacteria in response to nitrate exposure (from arugula juice) highlights their ecological adaptability.

Considering that smoking may increase the risk of developing periodontitis (34), it is important to evaluate if, in addition to health-associated oral bacteria (NRB), the addition of nitrate exogen affects the dysbiosis-associated periodontal pathogens (*P. gingivalis* and *F. nucleatum*). This study revealed that arugula nitrate may reduce the accumulation of periodontopathogen-associated dysbiotic bacteria, which was not observed previously when nitrate was added to a healthy community (35). Our *in vitro* study revealed an important finding: periodontopathic bacteria from non-smokers appeared to be larger in number than those from smokers in all biofilm conditions. This suggests that smoking-related dysbiosis may suppress these species in environments enriched with nitrate. In addition, anaerobic conditions tend to have greater bacterial proportions than aerobic ones, which highlights that periodontopathic bacteria grow in oxygen-limited situations (36).

Interestingly, *F. nucleatum* seems to outcompete *P. gingivalis* in aerobic conditions, particularly over 9 h and with higher nitrate concentrations. In contrast, under anaerobic conditions, especially after 9 h, both nitrate concentrations (3.25 and 6.25 µM) were effective in inhibiting the development of *P. gingivalis*. Again, the decrease was more noticeable in BS samples, which may be an indication of differences in converting nitrate to nitrite, leading to the susceptibility of smokers’ periodontopathic bacteria to environments containing NO. These findings support recent studies suggesting that some anaerobes related to periodontitis are vulnerable to oxidative stress, which renders them exposed to the antibacterial effects of NO (37–39). Since *F. nucleatum* plays a role in converting nitrate to nitrite and linking the aerobic and anaerobic niches (22, 40), the bacterium benefits from nitrite formation while maintaining strict anaerobic bacteria such as *P. gingivalis* (41). However, under aerobic conditions, the drastic nitric depletion in BS at 9 h may indicate increased nitrite utilization by *P. gingivalis* or other anaerobes as they adapt to oxygen stress. This behavior aligns with studies showing that *P. gingivalis* can metabolize nitrite under microaerophilic stress (42, 43). However, the mechanisms behind *P. gingivalis*'s higher sensitivity to nitric oxide than *F. nucleatum* and how these interactions could be used clinically to treat periodontal disease require further research, especially in light of the growth inhibition patterns observed, particularly in BS samples and after longer incubation periods.

According to our data, non-smokers’ oral bacteria may prefer anaerobic metabolism because of the changed oxygen tension in smokers’ mouths (44). This could lead to a decrease in the prevalence or metabolic activity of nitrate-reducing bacteria, as evidenced by the aforementioned decrease in salivary nitrate–nitric concentration. By referring to the results of the transcription levels of nitrate-associated genes, we found that in the presence of arugula nitrate (3.25 or 6.25 µM) and both anaerobic and aerobic environments, we found that non-smoking-associated bacteria may upregulate these genes more efficiently than smokers’ bacteria, leading to the nitrate-reducing bacteria being more metabolically active. This could indicate an *in vivo* event in which smoking promotes an alteration in the composition of oral bacteria, which are dominated by dysbiotic bacteria (45). Oxygen levels affect the pattern of *narG* and *napA* expression. While *narG* is often implicated in anaerobic reduction, *napA* is active in both anaerobic and aerobic environments and may act as an alternative mechanism when oxygen levels are low (45). Both genes’ higher transcription at 9 h, particularly in BNS, suggests that the microbiota in BNS is better able to adjust to nitrate availability, which improves nitrate metabolism in aerobic environments.

Taken together, our *in vitro* experiment suggests that more nitrate and anaerobic conditions encourage the growth of periodontopathic bacteria, especially *F. nucleatum*. These conditions are probably made possible by nitrate-reducing bacteria, which alter the biofilm environment to encourage anaerobiosis and resource availability. Because smokers’ biofilms have lower bacterial proportions, smoking-related dysbiosis appears to influence this interaction.

## 5 Limitations of this study

This study has some limitations. First, it was designed as a pilot *in vitro* investigation, thus, it might not fully capture the complexity of the oral environment *in vivo*. Given the pilot nature of this *in vitro* study and its focus on assessing the feasibility and preliminary effect of arugula juice on biofilm, a formal power analysis was not conducted. The sample size was deemed sufficient to establish the *in vitro* model and provide initial indications of potential effects. However, we acknowledge that this study was not designed to provide definitive conclusions about differences in the *in vivo* oral microbiome between smokers and non-smokers. Future studies with *in vivo* sampling and analysis would require a power analysis to determine the appropriate sample size for such a comparison. Additionally, the study focused solely on smokers and non-smokers, and the analysis was limited to a small number of distinct bacterial species. Finally, larger-scale *in vivo* studies are needed to confirm these findings and assess the clinical significance of arugula juice for smokers’ oral health. A more thorough picture would be obtained by analyzing the entire microbial community.

## 6 Conclusion

This *in vitro* study provides preliminary evidence that nitrate-rich arugula juice may benefit smokers’ oral health by supporting the growth of nitrate-reducing bacteria and possibly inhibiting periodontopathogens. However, further research is necessary to fully understand the complex relationships among nitrate, oxygen levels, bacterial species, and smoking-related dysbiosis. To confirm these results and ascertain the clinical significance and proper application of arugula juice as a potential therapy or preventive approach for strengthening smokers’ oral health, larger-scale *in vivo* research is essential.

## Data Availability

The raw data supporting the conclusions of this article will be made available by the authors, without undue reservation.
